# Rail Light-Strip Abnormality Analysis from Color Inspection Images Using an Improved SegFormer and Geometric Rules

**DOI:** 10.3390/s26165292

**Published:** 2026-08-21

**Authors:** Haoran Song, Yuntao Gou, Ning Wang, Le Wang, Junbo Liu, Shengchun Wang, Chengliang Xia, Qiang Han, Zichen Gu

**Affiliations:** 1 Infrastructure Inspection Research Institute, China Academy of Railway Sciences Corporation Limited, Beijing 100081, China; songhaoran@rails.cn (H.S.); gyt_rails@163.com (Y.G.); wangning2017@rails.cn (N.W.); 13001988989@163.com (L.W.); liujunbo@rails.cn (J.L.); wangshengchun@rails.cn (S.W.); solsoar@163.com (C.X.); hanqiang_iic@163.com (Q.H.); 2 INMAI Railway Technology Co., Ltd., Beijing 100081, China

**Keywords:** rail light strip, color inspection image, semantic segmentation, SegFormer, geometric rule, abnormality localization

## Abstract

**Highlights:**

**What are the main findings?**

- Boundary-enhanced SegFormer improves rail-head and light-strip mask recovery in color inspection images.
- Rail-head-constrained geometric rules identify eccentricity, width mutation and local integrity abnormalities.

**What are the implications of the main findings?**

- Boundary quality directly affects light-strip centerline and width measurements.
- Mask-derived geometric indicators provide interpretable evidence for rail maintenance analysis.

**Abstract:**

Rail light-strip morphology reflects the wheel-rail contact condition. Reliable automatic analysis remains difficult. The strip is narrow and has weak boundaries, while specular reflection, rail-head texture and trackside background interfere with color inspection images. This study proposes a segmentation-guided geometric method for rail light-strip abnormality analysis. An improved SegFormer jointly segments the background, rail-head and light-strip regions. A boundary detail enhancement module refines weak rail-head and light-strip contours. Focal Loss emphasizes minority and hard boundary pixels. The rail-head mask provides the geometric reference for extracting the light-strip centerline, eccentricity, width sequence and connected-component morphology. The predicted masks are ordered using the corrected mileage record. Every 1000 original-resolution rows then form a consecutive 1 m detection unit. When a geometric rule is triggered, the method reports that unit’s 1 m mileage interval together with its eccentricity, width-change or local-integrity measurement. The model achieves 95.67% mean Intersection over Union (mIoU) on 3520 annotated images. It detects 845 of 876 positive units, with 96.46% recall, 89.23% precision and 92.70% F1-score. The resulting records identify abnormal 1 m mileage intervals and report the corresponding eccentricity, width-change, or local-integrity measurements for targeted manual review.

## 1. Introduction

The rail head is the bearing and guiding interface between the wheel and the track. Repeated wheel-rail contact forms a continuous bright running band on its surface, referred to as the rail light strip in this study. The strip center, width and local contour morphology reflect contact location, grinding condition and local rail-surface state [1,2,3,4]. Rail condition assessment combines track-geometry measurement, ultrasonic or eddy-current inspection and visual observation. These techniques provide complementary information on geometry, internal flaws and surface condition. A comprehensive rail inspection vehicle is an onboard platform that synchronizes these measurements with visible-light images and corrected mileage during line inspection. Its imaging subsystem provides continuous, non-contact color records at operating speed. This allows a suspicious interval to be traced back to the corresponding image sequence. Recent studies have used color cameras, laser profilometers and three-dimensional range imaging to locate running surfaces or detect rail-surface defects [5,6,7]. The visible running band considered here is not equivalent to a crack or another isolated defect. Its condition is expressed mainly by its lateral position, width and contour relative to the rail head. Reliable analysis therefore requires both regions to be extracted in the same geometric reference. The analysis must also remain tied to acquisition mileage because isolated frame numbers are not useful to inspectors.

Current light-strip inspection still relies mainly on manual image review and local intensity or threshold-based extraction [2,3,4]. Manual review is time-consuming for continuous line-scan data. Brightness-based extraction is sensitive to specular reflection, stains, rail-head texture and trackside background. General rail-surface defect detectors are useful for cracks, spalling and other localized defects [1,6,7,8,9,10]. Recent computer-vision studies have also improved railway-component detection under limited data, rail-surface defect detection through feature fusion and running-surface measurement under vehicle-based sensing [5,10,11]. However, their outputs are generally component boxes, defect categories, defect masks or laser-derived surface measurements. They do not directly convert color-image segmentation of the light strip and rail head into mileage-linked eccentricity, width-change and local-integrity indicators. A stable solution must preserve the weak boundaries of both regions and use the rail head as the reference for downstream measurements. Unlike image-level classification, the expected output must answer whether an abnormality occurs, where it is located and which geometric change causes the alarm. The proposed method therefore combines a boundary-enhanced three-class SegFormer with rail-head-constrained geometric rules. Its contribution is the coupling of boundary-sensitive segmentation and explicit geometric abnormality criteria, rather than segmentation alone. This design yields a 1 m mileage interval and the measured condition that triggered each abnormality record.

The remainder of this paper is organized as follows. Section 2 reviews vision-based rail inspection and semantic segmentation methods. Section 3 describes the improved SegFormer, geometric parameters and abnormality rules. Section 4 presents the dataset, implementation details and experimental results. Section 5 discusses practical implications and limitations, and Section 6 concludes the paper.

## 2. Related Work

Vision-based railway inspection has progressed from handcrafted features and template matching to deep networks capable of handling scale variation and cluttered trackside scenes [1,2,3,4]. Recent rail defect detectors and industrial surface-segmentation methods improve small-defect representation, class imbalance and pixel confidence [1,2,8,9]. However, these methods mainly identify cracks, spalling, scratches or other local defects. They do not explicitly describe the relative geometry between the wheel-rail running band and the rail head.

Semantic segmentation has evolved from convolutional encoder–decoder models and multiscale context aggregation [12,13,14,15] to transformer architectures with stronger long-range modeling [16,17,18,19]. Boundary-oriented methods commonly introduce an independent shape stream, model-agnostic boundary correction or boundary-sensitive evaluation [20,21,22]. Other context and edge-enhancement approaches improve semantic consistency or fine boundary recovery [23,24,25,26,27,28], while promptable segmentation models provide strong generic masks [29]. These developments confirm the importance of boundary quality when downstream measurements depend on narrow or elongated structures.

The proposed boundary detail enhancement module differs from a separate high-resolution shape stream and from post-processing that corrects an already generated mask. It is attached to the SegFormer decoder and fuses shallow boundary-sensitive features with high-level semantic features through channel alignment and an edge-aware spatial weight. This compact refinement is suited to rail light strips because their transverse width is small and their longitudinal structure is continuous. Small boundary displacements are amplified in subsequent centerline and width calculations. Thus, the study evaluates segmentation as both pixel classification and the geometric basis for rail-head-constrained abnormality analysis.

## 3. Materials and Methods

### 3.1. Overview of the Proposed Method

**Figure 1:**
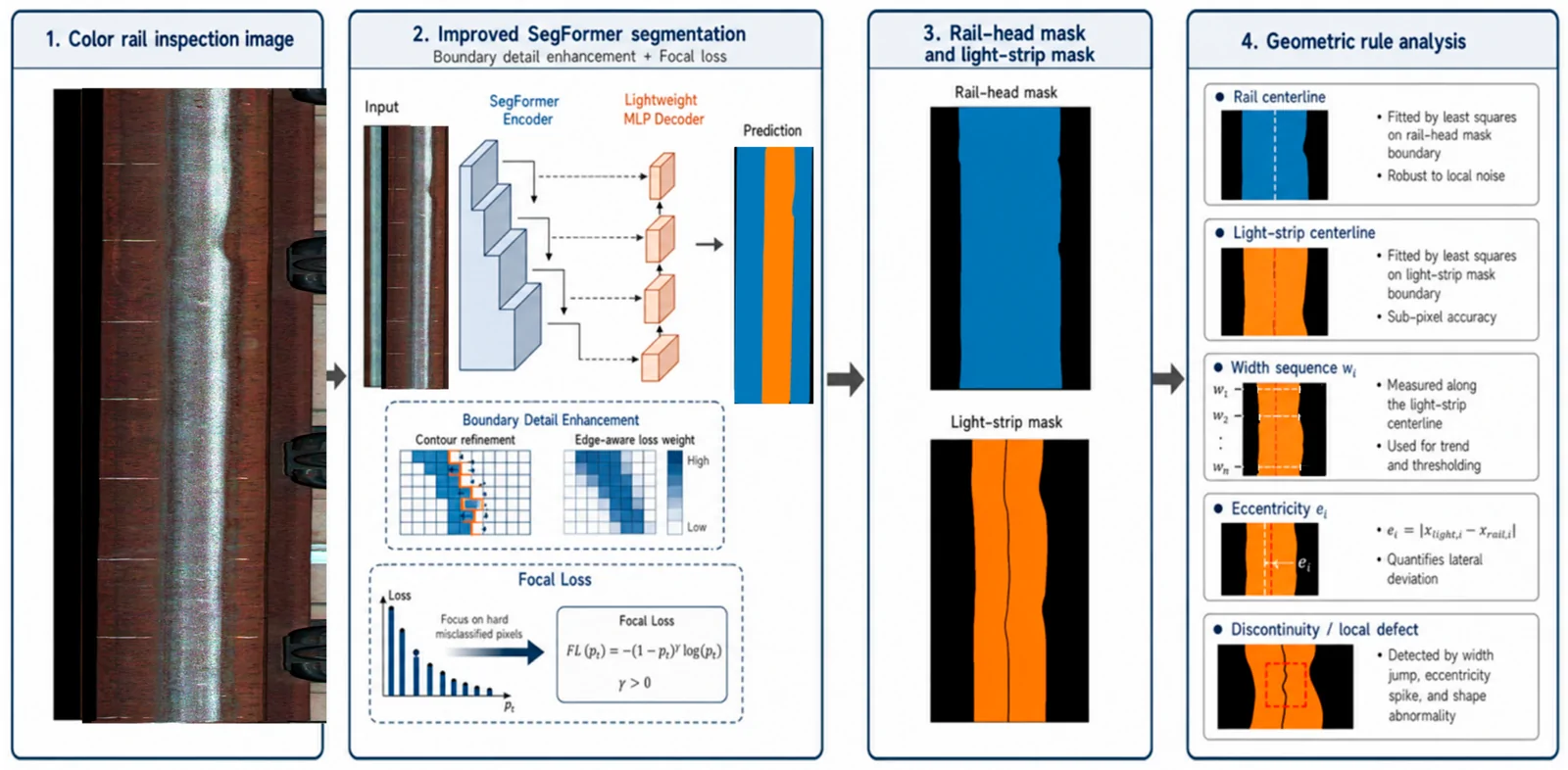
Overview of the proposed rail light-strip abnormality analysis method.

Figure 1 summarizes the segmentation-guided analysis pipeline. The color image is first divided into background, rail-head and light-strip regions. The rail-head mask defines the geometric reference, while the light-strip mask preserves the contact-trace morphology. The rule module then answers three operational questions. It identifies the mileage location, measures the strip shift relative to the rail-head centerline and describes width or local-contour changes. The outputs are therefore a location and measurable geometric indicators, rather than only a pixel mask.

### 3.2. Improved SegFormer for Rail-Head and Light-Strip Segmentation

SegFormer is used because its hierarchical Mix Transformer (MiT) encoder models long-range dependencies while retaining multiscale features [19]. The MiT-B2 variant is adopted for three-class segmentation of the background, rail head and light strip. A lightweight multilayer perceptron (MLP) decoder produces the initial prediction. The boundary detail enhancement module then refines the prediction before geometric analysis.

The input image is first encoded into multilevel feature maps. Low-level features preserve texture, brightness transition and boundary information, whereas high-level features provide more stable semantic context for distinguishing the rail head from the background. The decoder fuses these features and outputs a three-channel prediction map corresponding to the background, rail-head region and light-strip region. Compared with binary light-strip extraction, this three-class setting is more suitable for geometric analysis because the rail-head mask provides the reference frame for interpreting the light-strip position.

Weak or blurred boundaries are common in reflective rail images. The boundary detail enhancement module explicitly combines shallow texture information with the semantic feature produced by the SegFormer decoder. The two inputs are first aligned by 1 × 1 convolutions. Their concatenation is then processed by a boundary-guidance branch to generate a spatial weight map. This map enhances the semantic feature around rail-head and light-strip contours. Finally, the weighted semantic feature is concatenated with the aligned shallow feature and refined by a 3 × 3 convolution. The Gated Shape Convolutional Neural Network (Gated-SCNN) maintains a parallel shape stream [20]. SegFix corrects pixels after mask prediction [21]. By contrast, the proposed module performs feature refinement inside the decoder.

For an input image of H×W pixels, the MiT-B2 encoder outputs four feature maps $F_{1},F_{2},F_{3},F_{4}$ with channel numbers [64, 128, 320, 512] and nominal resolutions [$\frac{H}{4}\times \frac{W}{4},\frac{H}{8}\times \frac{W}{8},\frac{H}{16}\times \frac{W}{16},\frac{H}{32}\times \frac{W}{32}$]. Each feature map is projected to 256 channels by the MLP decoder. $F_{1}$ is retained at the stage-1 resolution. $F_{2}$, $F_{3}$ and $F_{4}$ are bilinearly resized with nominal factors of 2, 4 and 8, respectively. The resizing operation explicitly uses the $F_{1}$ spatial size, with align_corners set to false, to avoid mismatches caused by an odd image width. The aligned maps are concatenated in the order [$F_{1}$, $Up\left(F_{2}\right)$, $Up\left(F_{3}\right)$, $Up\left(F_{4}\right)$] to form a 1024-channel tensor. A 1 × 1 convolution followed by batch normalization and ReLU reduces it to the 256-channel decoder feature $F_{d}$.

The shallow input of the boundary module is the stage-1 feature $F_{1}$, and the high-level input is $F_{d}$. A 1 × 1 convolution maps $F_{1}$ from 64 to 128 channels, and another 1 × 1 convolution maps $F_{d}$ from 256 to 128 channels. Both tensors therefore have 128 channels at $\frac{H}{4}\times \frac{W}{4}$. They are concatenated in the order [$F_{d}$, $F_{1}$]. The concatenated tensor passes through a 3 × 3 convolution from 256 to 128 channels, followed by batch normalization and ReLU. A 1 × 1 convolution then maps 128 channels to 1 channel. Sigmoid converts the result into the boundary weight map. The aligned semantic feature is multiplied by one plus this weight map. It is then concatenated with the aligned $F_{1}$ feature. A final 3 × 3 convolution reduces the 256-channel tensor to a 64-channel refined feature. A 1 × 1 classifier produces three class logits, which are bilinearly resized to the input size.

With the 512 × 994 input used in the experiments, $F_{1},F_{2},F_{3},F_{4}$ have spatial sizes 128 × 249, 64 × 125, 32 × 63 and 16 × 32, respectively. All decoder features are resized to 128 × 249 before fusion. The boundary module outputs a 64 × 128 × 249 refined tensor and a 3 × 128 × 249 logit tensor. The final prediction is resized directly to 3 × 512 × 994. The boundary branch adds approximately 0.48 M trainable parameters, which is consistent with the 27.4 M to 27.9 M increase evaluated in Section 4.4.

(1) P=Softmax(Dθ(Eθ(I))),M^=argmaxPk

Here, I denotes the input color rail image. $E_{\theta }$ and $D_{\theta }$ denote the SegFormer encoder and decoder. P is the class probability map. k indexes the semantic class. M^ is the predicted segmentation mask.

(2) $$\begin{array}{c} A=\sigma \left(C_{1\times 1},\left(C_{3\times 3},\left[F_{s},F_{l}\right]\right)\right)\\ F_{b}=C_{3\times 3}\left[F_{s}⊙\left(1+A\right),F_{l}\right] \end{array}$$

Here, $F_{s}$ and $F_{l}$ are the 128-channel aligned semantic and shallow features, respectively. A is the single-channel boundary weight map. $C_{k\times k}$ denotes a convolution with kernel size k×k. σ is the sigmoid function. $F_{b}$ is the 64-channel boundary-refined feature used by the final three-class classifier.

**Figure 2:**
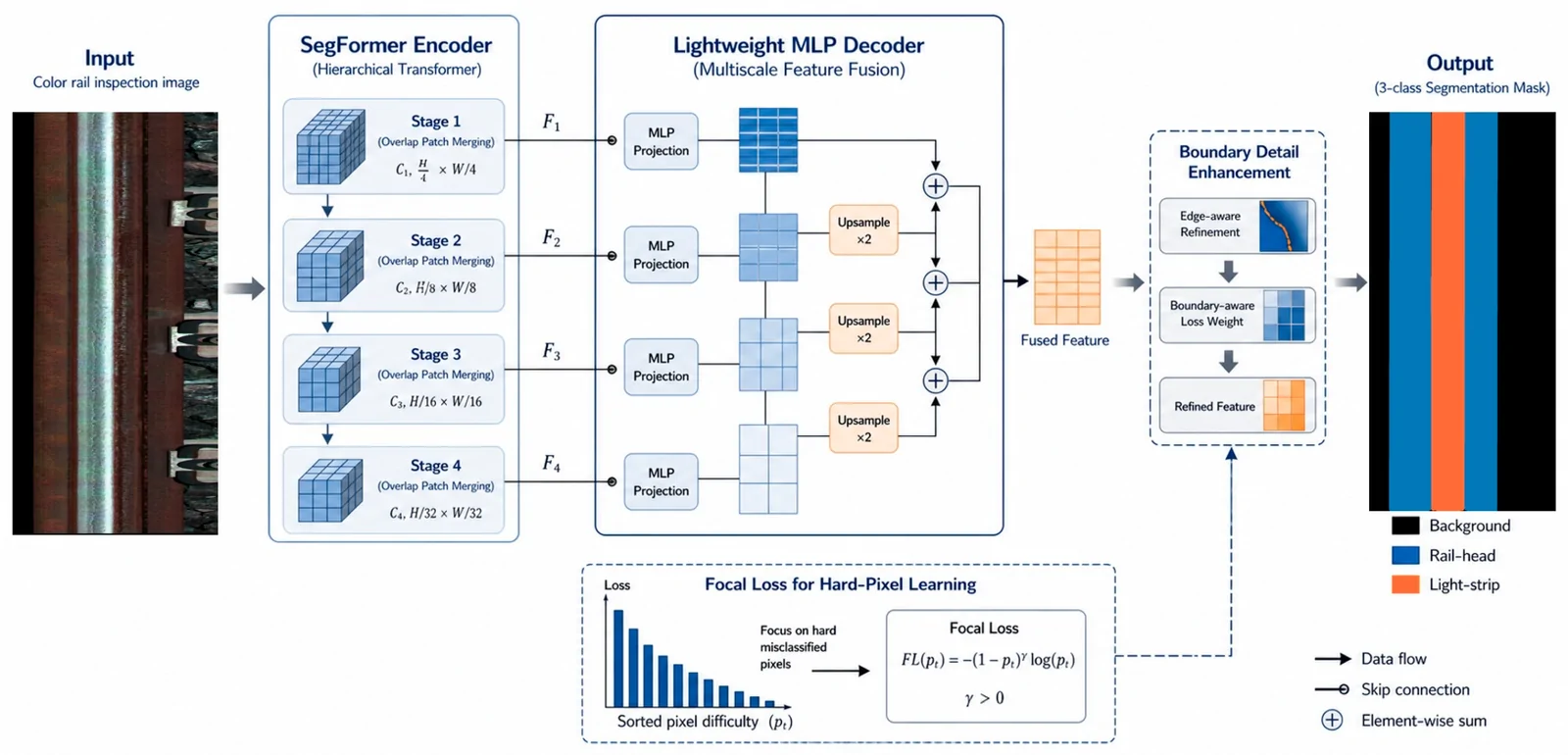
Architecture of the improved SegFormer for rail-head and light-strip segmentation, including the hierarchical encoder, lightweight MLP decoder, boundary detail enhancement module and Focal Loss.

**Figure 3:**
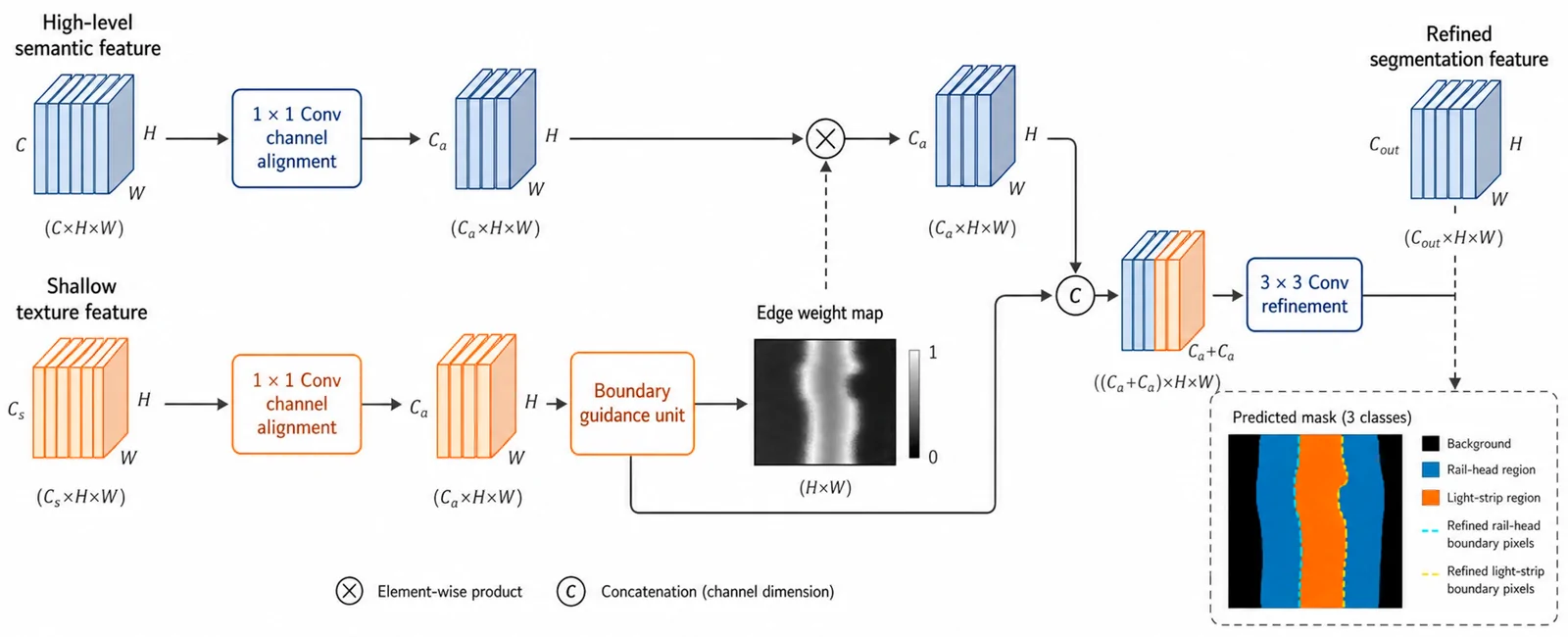
Boundary detail enhancement module used in the improved SegFormer.

Focal Loss is further used during training to reduce the dominance of well-classified background pixels [30]. This setting corresponds to the loss component in Figure 2 and complements the boundary enhancement module in Figure 3. Most background pixels are classified with high confidence, whereas rail-head and light-strip pixels near weak or reflective boundaries are more difficult. The modulating factor in Focal Loss suppresses the loss contribution of these easy background pixels. Consequently, hard rail-head and light-strip pixels receive relatively greater optimization attention, which improves the stability of the boundaries used by the subsequent geometric measurements.

(3) $$L_{focal}=-\frac{1}{N}\sum_{n=1}^{N}\sum_{k=1}^{K}\alpha_{k}{\left(1-p_{n,k}\right)}^{\gamma }y_{n,k}log(p_{n,k})$$

Here, N is the number of pixels. K is the number of classes. $y_{n,k}$ is the one-hot label. $p_{n,k}$ is the predicted probability. $\alpha_{k}$ was set to 0.25 for all three classes, and the focusing parameter γ was set to 2.0. The focusing term reduces the contribution of well-classified pixels rather than assigning different fixed weights to the three classes.

The improved structure retains the SegFormer encoder–decoder topology and adds only the boundary-refinement branch and Focal Loss. Section 4.4 evaluates its computational overhead. The baselines are DeepLab v3+, Segmenter, the Object-Contextual Representations Network (OCRNet) and the Bilateral Segmentation Network V2 (BiSeNetV2). They represent convolutional encoder–decoder segmentation, transformer segmentation, object-context modeling and real-time bilateral segmentation, respectively [15,17,24,25].

### 3.3. Geometric Rule-Based Abnormality Analysis

**Figure 4:**
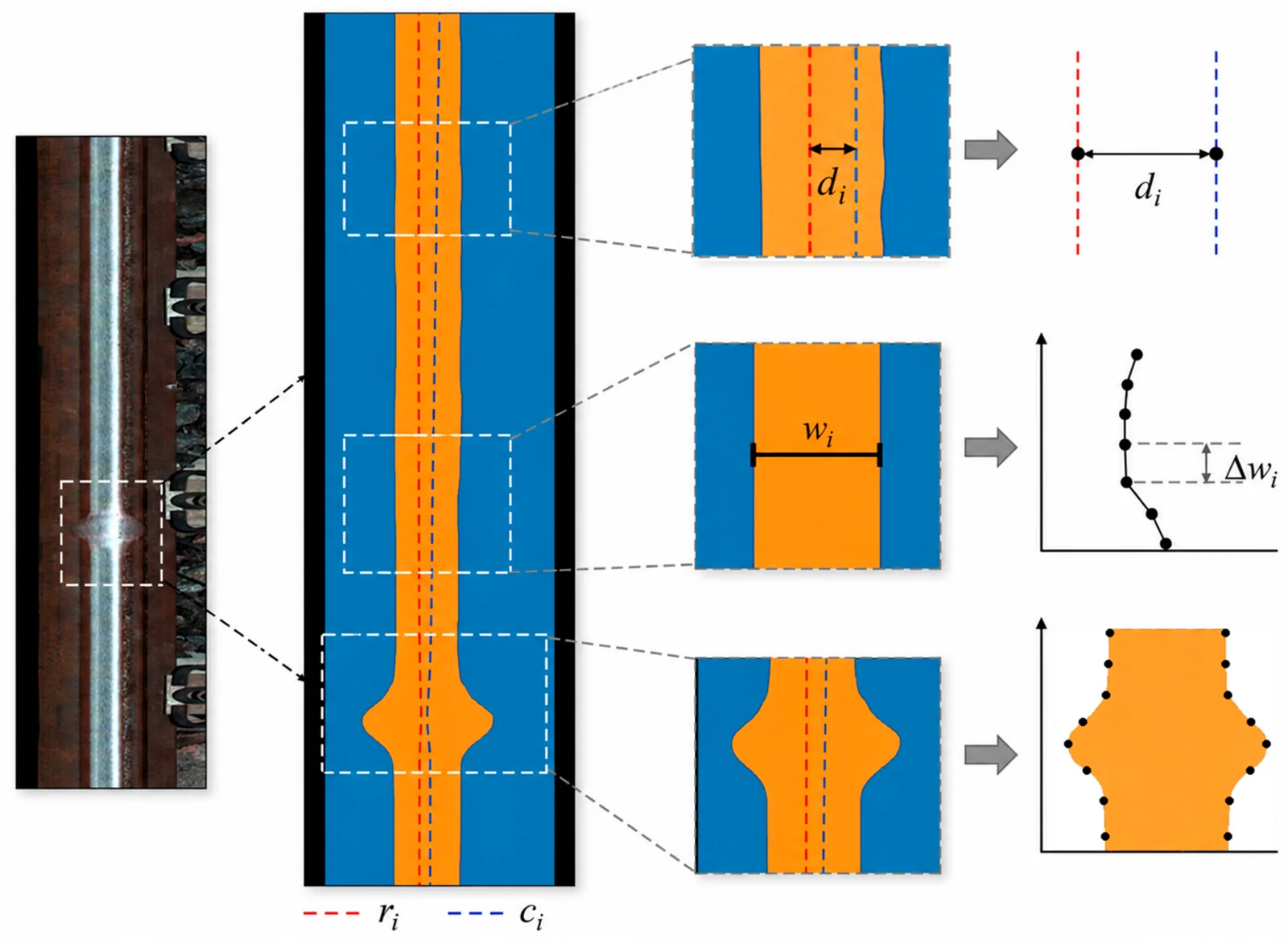
Mask-derived geometric parameters used for rail light-strip abnormality analysis.

After obtaining the two masks, the prediction is restored from 512 × 994 pixels to the original resolution of 1024 × 1988 pixels by nearest-neighbor interpolation. The rail-head boundaries are then fitted, and their midpoint defines the rail centerline. The light-strip boundaries are sampled along the rail direction to obtain center position, width and local contour morphology. These quantities are associated with the corrected mileage record. Each output can therefore be traced to a consecutive 1 m detection unit, as illustrated in Figure 4.

The original longitudinal sampling resolution is 1 mm per image row. Therefore, the 1988 rows in one original image cover approximately 1.988 m of rail. Consecutive masks are ordered and concatenated according to the encoder-triggered and system-corrected mileage record. Every 1000 rows at the restored original resolution form one 1 m detection unit, and a unit may cross an image boundary. For row y in unit i, the left and right rail-head boundaries are denoted by $x_{L}^{r}$ and $x_{R}^{r}$, and the light-strip boundaries by $x_{L}^{s}$ and $x_{R}^{s}$. The row-wise light-strip width is $b\left(y\right)=k_{x}\left(x_{R}^{s}\left(y\right)-x_{L}^{s}\left(y\right)\right)$. Row-wise center positions and widths are aggregated by their median values within each 1 m unit to suppress isolated fluctuations caused by reflection and noise.

(4) $$\begin{array}{c} r_{i}=median\left(\frac{x_{L}^{r}+x_{R}^{r}}{2}\right),c_{i}=median\left(\frac{x_{L}^{s}+x_{R}^{s}}{2}\right)\\ B_{i}=k_{x}median\left(x_{R}^{s}-x_{L}^{s}\right) \end{array}$$

Here, $r_{i}$ and $c_{i}$ are the rail-head and light-strip center positions in the i-th 1 m unit, respectively. $B_{i}$ is the corresponding light-strip width. $k_{x}$ is the horizontal image calibration coefficient in mm/pixel.

(5) $$e_{i}=k_{x}\left(c_{i}-r_{i}\right),{\Delta B}_{i}=\left|B_{i}-B_{i-1}\right|$$

Here $e_{i}$ is the signed lateral eccentricity of the light strip relative to the rail-head centerline, and ${\Delta B}_{i}$ is the absolute width change between adjacent 1 m detection units. The eccentricity rule uses the absolute value $\left|e_{i}\right|$ in each unit rather than an adjacent-unit eccentricity difference.

(6a) $$D_{i}=\underset{\Omega \subset U_{i},\left|\Omega \right|=100}{max}\left|\underset{y\in \Omega }{median}b\left(y\right)-B_{i}\right|$$

(6b) $$m_{i}=1\left(D_{i}>10\;mm\wedge L_{i}\geq 100\;mm\wedge {\Delta B}_{i}\leq 10\;mm\right)$$

(6c) $$A_{i}=1\left(\left|e_{i}\right|>6\;mm\right)+1\left({\Delta B}_{i}>10\;mm\right)+m_{i}$$

In Equation (6a–c), $U_{i}$ is the set of 1000 original-resolution rows in the i-th detection unit. Ω is a 100-row sliding window that corresponds to 100 mm in the longitudinal direction. $D_{i}$ is the maximum deviation between the local median width and the 1 m median width $B_{i}$. $L_{i}$ is the longitudinal length covered by the merged abnormal windows. The binary indicator $m_{i}$ is activated only when a local width deviation exceeds 10 mm for at least 100 mm, and the adjacent-unit width-mutation rule is not triggered. Eccentricity abnormality is a positional condition and may coexist with local contour deformation. $A_{i}$ counts the triggered abnormality conditions, and a unit with $A_{i}>0$ is reported for manual review.

An eccentricity abnormality is reported when $\left|e_{i}\right|>6\;mm$, and a width mutation is reported when ${\Delta B}_{i}>10\;mm$. A local integrity abnormality is reported when $m_{i}=1$. It describes a short-range expansion, contraction or unilateral contour deformation within a 1 m unit, rather than an overall width change between adjacent 1 m units. The condition ${\Delta B}_{i}\leq 10\;mm$ in Equation (6b) prevents the local-integrity category from duplicating width mutation. Eccentricity measures position, whereas local integrity measures contour morphology. Both indicators may therefore be activated in the same unit. The manually audited primary label is used for per-type statistics.

The rule thresholds are engineering mappings rather than parameters optimized on the validation set. DB34/T 3964-2021 specifies a 6 mm light-strip center offset [31]. For a local region longer than 100 mm, it also specifies an approximately 10 mm width difference relative to the overall mean [31]. The 6 mm value is used as the absolute eccentricity limit. The 10 mm value is used for both adjacent-unit width mutation and the local width-deviation test. The 100 mm local-window length follows the minimum longitudinal extent in the same local-abnormality requirement. The high-speed railway maintenance rule also identifies poor contact bands by widths below 20 mm or above 40 mm and by periodic width variation [32]. The 200 mm association threshold for neighboring local contour regions follows the criterion for adjacent surface damage in railway maintenance practice [33]. All rule calculations use masks restored to the original resolution. The longitudinal scale is 1 mm per row, and the calibrated horizontal scale is $k_{x}=0.2\;mm/pixel$.

The geometric-rule hyperparameters are defined accordingly. They comprise a 6 mm absolute-eccentricity threshold, a 10 mm width-change threshold, a 100 mm local-window length and a 200 mm association distance. These values are applied after image measurements are converted to physical units. They remain identical for every segmentation model.

Longitudinal geometric sequences reduce alarms caused by isolated brightness changes. Reflection or stains may create high local contrast without a coherent centerline displacement, width change or contour deformation. A real abnormality is expected to persist spatially or produce a connected contour change. The rule module therefore evaluates local measurements together with connected-component morphology.

Each abnormal output record contains the 1 m start-end mileage interval, abnormality type, eccentricity, width change and local-integrity flag. The reported interval follows the corrected mileage sequence used to order and concatenate the masks. Therefore, a triggered unit can be traced to the corresponding inspection segment even when it crosses an image boundary. These are termed maintenance-related indicators because they can direct personnel to a specific interval for manual review. They do not constitute a maintenance grade or an automatic maintenance decision.

## 4. Experiments and Results

### 4.1. Dataset and Experimental Settings

The dataset contains 3520 color visible-light images acquired on multiple railway lines. Each original image has a resolution of 1024 × 1988 pixels. Pixel labels include background, rail head and light strip and are stored in the PASCAL Visual Object Classes (VOC) 2012 format [34,35]. The dataset was divided into 2464 training images and 1056 validation images at a ratio of 7:3. Splitting was performed by railway line and continuous acquisition section. Images from the same section and adjacent frames from the same sequence were assigned to only one subset, preventing section-level leakage.

The 1056-image validation set contained 876 manually audited ground-truth-positive 1 m units. These comprised 438 width-mutation, 292 eccentricity-abnormality and 146 local integrity abnormality units. The mutually exclusive primary labels are used for the per-type abnormality statistics. The counts represent detection units rather than images. Each 1024 × 1988 image covers approximately 1.988 m, and a 1 m unit may cross an image boundary. Negative validation units are not included in the 876 positive count but are retained when counting false positives. The abnormality labels are not additional semantic classes because normal and abnormal images share the same three-pixel categories. The complete dataset cannot be publicly released because it contains proprietary railway inspection data. Authorized access may be considered upon reasonable request.

Ground-truth masks were generated through human interactive annotation. Annotators first used interactive prompts to obtain the initial rail-head and light-strip regions. They then refined the contours manually at the pixel level, focusing on weak, reflective and locally deformed boundary areas. The refined masks were subsequently reviewed against the original color images. This review ensured consistent separation of the background, rail head and light strip.

The rail-surface imaging component was the GX3-LSM-02KGC-01A module (INMAI Railway Technology Co., Ltd., Beijing, China). It integrates a color line-scan camera, three-wavelength laser illumination, drive electronics, thermal management and a protective enclosure. The camera was a Linea LA-GC-02K05B color complementary metal-oxide-semiconductor (CMOS) line-scan camera (Teledyne DALSA, Waterloo, ON, Canada) with a 2048 × 2 pixel sensor layout and a pixel size of 7.04 × 7.04 μm. After region cropping, the effective line frequency was 45 kHz. Because the module uses line-scan imaging, line frequency, rather than area-camera frame rate, determines the longitudinal sampling capacity.

The camera was equipped with an LM25HC lens (Kowa Optronics Co., Ltd., Tokyo, Japan) with a focal length of 25 mm and an aperture of f/5.6. The nominal focusing distance from the module optical exit to the rail surface was 350 mm, and the specified working-distance range was 350–650 mm. Accordingly, 350 mm was used as the nominal installation-height reference. The configured camera field of view was 31.99°. The mounting angle in the vehicle-motion direction was adjustable from −8° to +8°, while the laser projection angle was 56°. The illumination combined 450, 520 and 650 nm laser bands to produce a white-light color image.

The camera accepted a 5–12 V external trigger through Line1 or Line2, and the laser source was synchronized by the camera trigger. This configuration allowed the rail head, bright light strip and surrounding fastener or ballast to be recorded in the same image. The fixed optical geometry also kept the rail-head and light-strip regions approximately aligned, providing a stable basis for annotation and geometric calibration.

**Figure 5:**
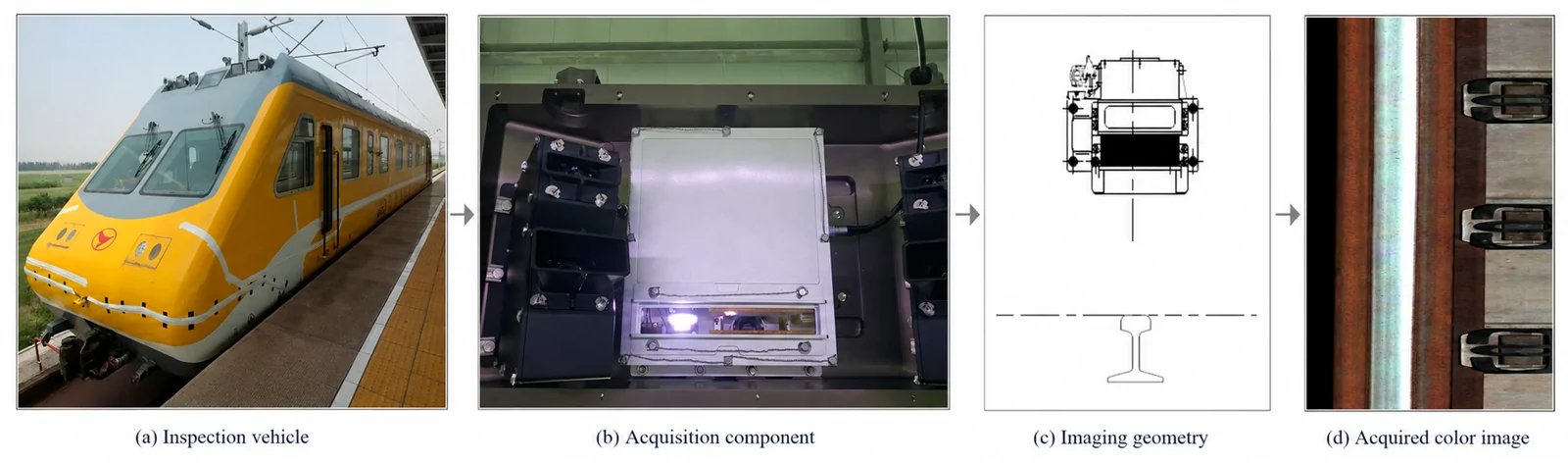
Rail light-strip image acquisition setup. (**a**) Comprehensive inspection vehicle; (**b**) visible-light acquisition component; (**c**) imaging geometry between the acquisition component and rail head; (**d**) representative acquired color rail image.

The maximum inspection speed was 160 km/h. Image acquisition was triggered by the encoder, and the recorded position was corrected using the mileage information provided by the integrated inspection system. This mechanism maintained the correspondence between each line-scan image and its railway mileage under varying vehicle speeds. The acquisition system and imaging geometry are shown in Figure 5.

The experimental platform used an Intel Xeon 2.4 GHz central processing unit (CPU), an NVIDIA RTX 3090 graphics processing unit (GPU) and 256 GB of memory. The software environment comprised Ubuntu 20.04, Python 3.10, PyTorch 2.4 and Compute Unified Device Architecture (CUDA) 11.8. ImageNet-pretrained MiT-B2 parameters initialized the network [36]. During validation and inference, each 1024 × 1988 image was deterministically downsampled by 0.5 to 512 × 994 pixels.

Training samples were processed in the following order: image loading, annotation loading, RandomResize, RandomCrop, RandomFlip, PhotoMetricDistortion and PackSegInputs. RandomResize used the base scale (1024, 512), a ratio range of 0.5–2.0 and aspect-ratio preservation. RandomCrop used a crop size of 512 × 512 pixels and a maximum single-class ratio of 0.75. RandomFlip was applied with a probability of 0.5. PhotoMetricDistortion used the default brightness delta of 32, contrast range of 0.5–1.5, saturation range of 0.5–1.5 and hue delta of 18. No random rotation was used.

The model was trained for 40,000 iterations using stochastic gradient descent. The initial learning rate was 0.01, momentum was 0.9 and weight decay was 0.0005 [37]. Focal Loss used α=0.25 and γ=2.0. A polynomial schedule with power 0.9 followed a 1500-iteration linear warm-up.

The physical mini-batch size was 8, with gradient accumulation over eight steps for an effective batch size of 64. The random seed was 42. Validation was performed every 1000 iterations. The checkpoint with the highest validation mIoU was selected, with light-strip IoU used as the tie-breaker. Preliminary training was used to finalize the learning rate, warm-up schedule, batch configuration and Focal Loss parameters. The criteria were loss convergence, validation stability and the memory capacity of the RTX 3090 platform. These settings control model optimization and are not used as thresholds in the geometric abnormality decision.

Evaluation is performed at three levels. Class-wise Intersection over Union (IoU) and mean Intersection over Union (mIoU) assess the three masks. Boundary F1-score (BF1) measures contour recovery within a 3-pixel tolerance [22]. Centerline mean absolute error (CL-MAE) and width mean absolute error (W-MAE) measure the geometric error propagated from segmentation. Recall, precision and F1-score assess the final abnormality labels obtained by applying the same rules to every segmentation model. The metrics are defined below.

(7) $${IoU}_{k}=\frac{{TP}_{k}}{{TP}_{k}+{FP}_{k}+{FN}_{k}}$$

(8) $$mIoU=\frac{1}{K}\sum_{k=1}^{K}{IoU}_{k}$$

(9) $$BF1=\frac{2P_{b}R_{b}}{P_{b}+R_{b}}$$

(10) $$CL-MAE=\frac{1}{N}\sum_{i=1}^{N}\left|c_{i}^{pred}-c_{i}^{gt}\right|$$

(11) $$W-MAE=\frac{1}{N}\sum_{i=1}^{N}\left|w_{i}^{pred}-w_{i}^{gt}\right|$$

(12) $$Recall=\frac{TP}{TP+FN},Precision=\frac{TP}{TP+FP},F1=\frac{2\times Precision\times Recall}{Precision+Recall}$$

In Equations (7)–(12), the intersection and union pixel counts are computed for each of the three classes. Boundary precision and boundary recall use a 3-pixel matching tolerance. N is the number of valid longitudinal sampling rows. The predicted center and width are compared with ground truth. For abnormality analysis, true positives (TP), false positives (FP) and false negatives (FN) are counted from the geometric-rule outputs.

### 4.2. Segmentation Results

All ablation experiments were evaluated on the same validation set under identical training and inference settings. Compared with the baseline SegFormer, the boundary enhancement module increased mIoU by 1.08 percentage points and improved BF1 from 64.72% to 65.43%. Meanwhile, CL-MAE decreased from 1.80 to 1.15 px, and W-MAE decreased from 5.61 to 4.63 px. This indicates improved boundary localization and geometric measurement accuracy. Focal Loss mainly improved light-strip segmentation, increasing LS IoU from 93.88% to 94.76%. Using both components produced the best overall performance. The model reached 95.67% mIoU and 65.75% BF1. CL-MAE and W-MAE were lowest at 1.11 and 4.50 px, respectively. These results demonstrate the complementary effects of boundary refinement and hard-pixel learning. The ablation results are summarized in Table 1.

**Figure 6:**
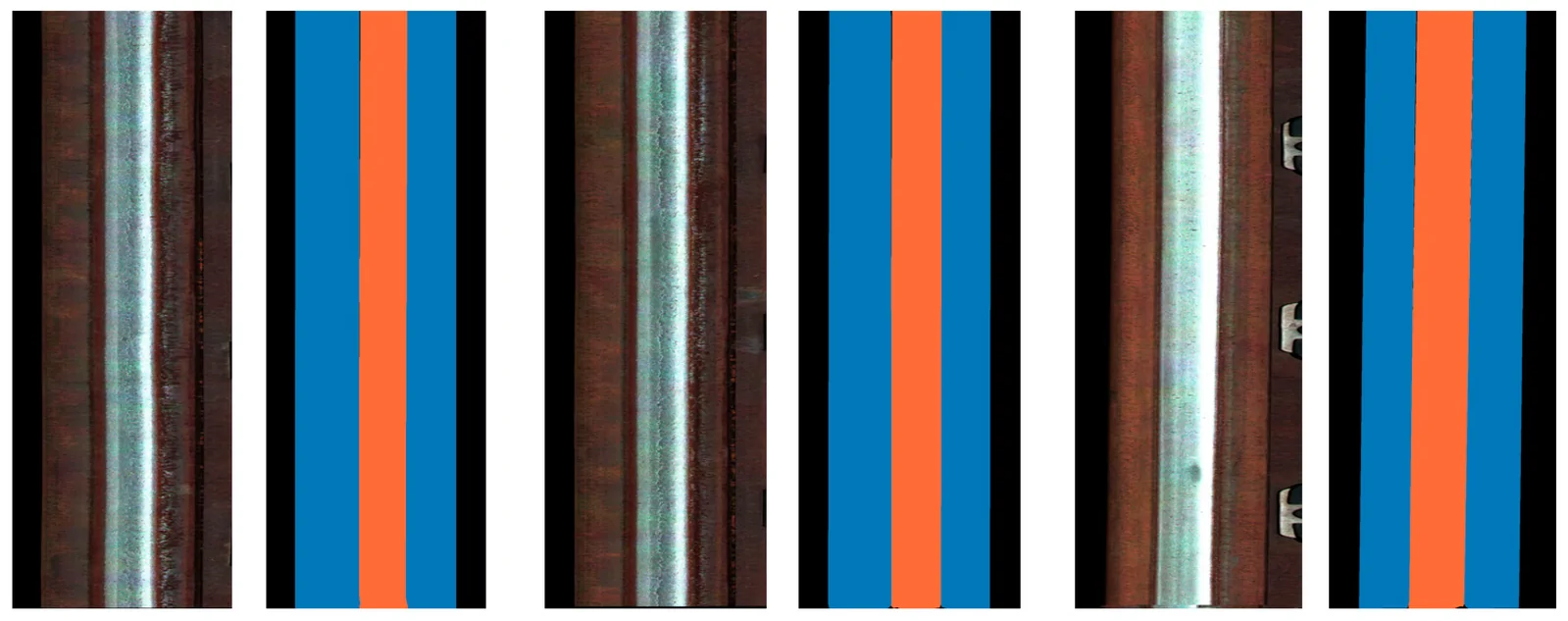
Segmentation results of the improved SegFormer on color rail inspection images. The left column shows original image patches and the right column shows corresponding masks.

Figure 6 further illustrates the segmentation behavior. For normal samples, the rail-head mask remains continuous, and the light-strip mask preserves the elongated contact-band morphology. Stable masks are necessary because a fragmented reference or strip contour directly disturbs the centerline and width sequence.

### 4.3. Abnormality Analysis Results

**Figure 7:**
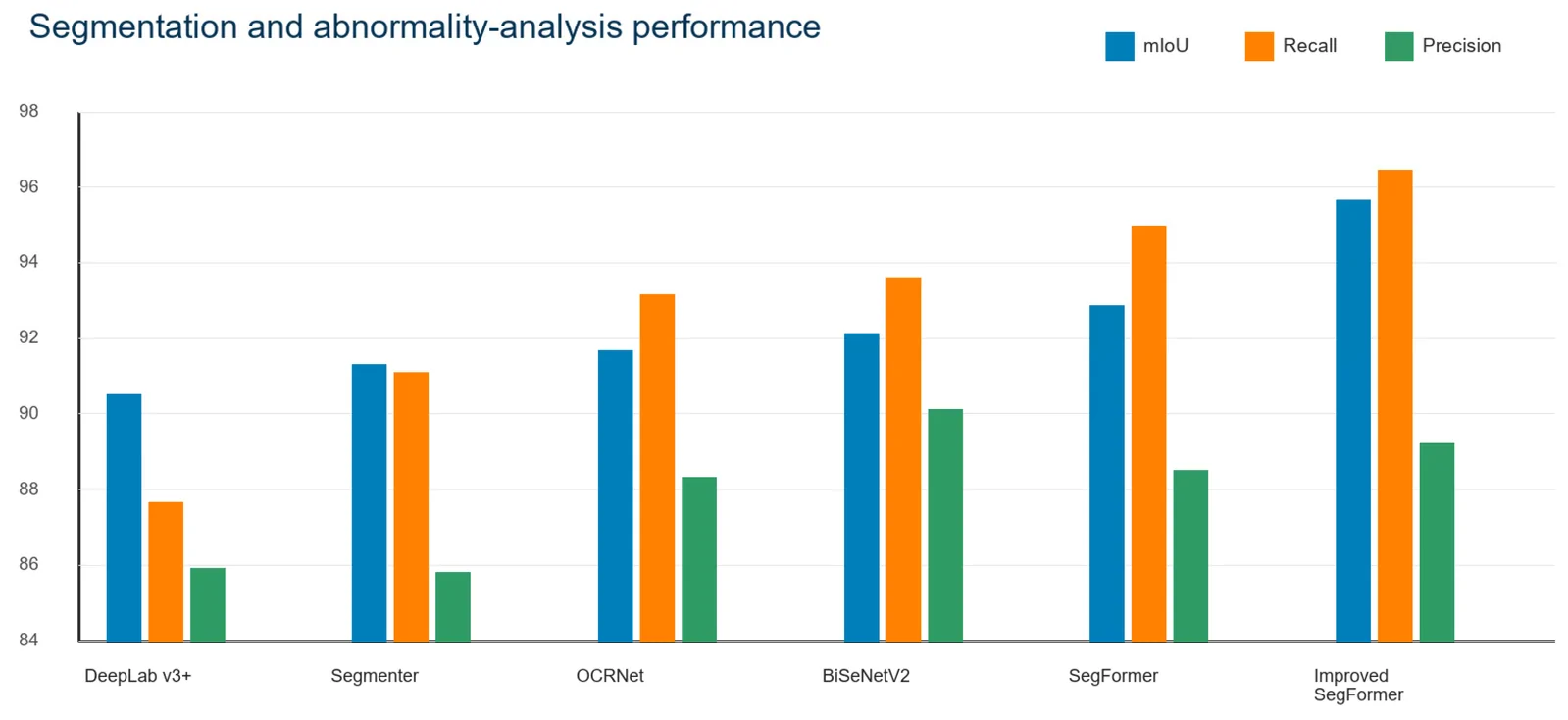
Comparison of segmentation and abnormality-analysis performance.

BiSeNetV2 yields the highest precision of 90.11%, whereas the proposed model achieves 89.23%. The proposed model nevertheless provides the highest mIoU (95.67%), recall (96.46%) and F1-score (92.70%) among the compared methods. Relative to the original SegFormer, it improves mIoU by 2.81 percentage points, recall by 1.48 points and F1-score by 1.07 points. This indicates a better balance between missed and false abnormality detections. The comparative results are summarized in Table 2 and visualized in Figure 7.

Segmentation accuracy and abnormality-analysis performance are related but not identical. Similar mIoU values can lead to different recall or precision because the rule module depends on boundary stability and local mask shape. A small error on the light-strip boundary can change the measured eccentricity or width more than the same number of background pixels.

For each 1 m unit, the geometric module records the start-end mileage interval, triggered rule and corresponding eccentricity, width change and local integrity result. Consecutive masks are ordered using the encoder-triggered and system-corrected mileage record. Therefore, a triggered unit is localized to its associated 1 m mileage interval, including a unit that crosses an image boundary. A record with one or more triggered conditions is flagged for targeted manual review. Table 3 quantifies the abnormality decisions. The flagged records are used to prioritize the corresponding 1 m mileage intervals for manual review, without directly assigning maintenance grades or initiating automatic maintenance actions.

The validation set contained 876 ground-truth-positive 1 m units for geometric-rule evaluation. Each unit received one mutually exclusive primary label: width mutation, eccentricity abnormality or local integrity abnormality. A unit with multiple geometric manifestations was assigned the manually confirmed primary abnormality label for per-type counting. Negative validation units were retained when calculating false-positive counts. Table 3 reports the resulting TP, FP and FN counts.

Among the three categories, width mutation achieved the highest recall (98.17%) and precision (91.10%). Local integrity abnormality was more challenging, with 93.15% recall and 86.08% precision. Its local area changes and irregularly connected contours are more sensitive to reflection, stains and weak boundaries. Across all 876 positive units, 845 were correctly detected. The overall recall, precision and F1-score were 96.46%, 89.23% and 92.70%, respectively.

**Figure 8:**
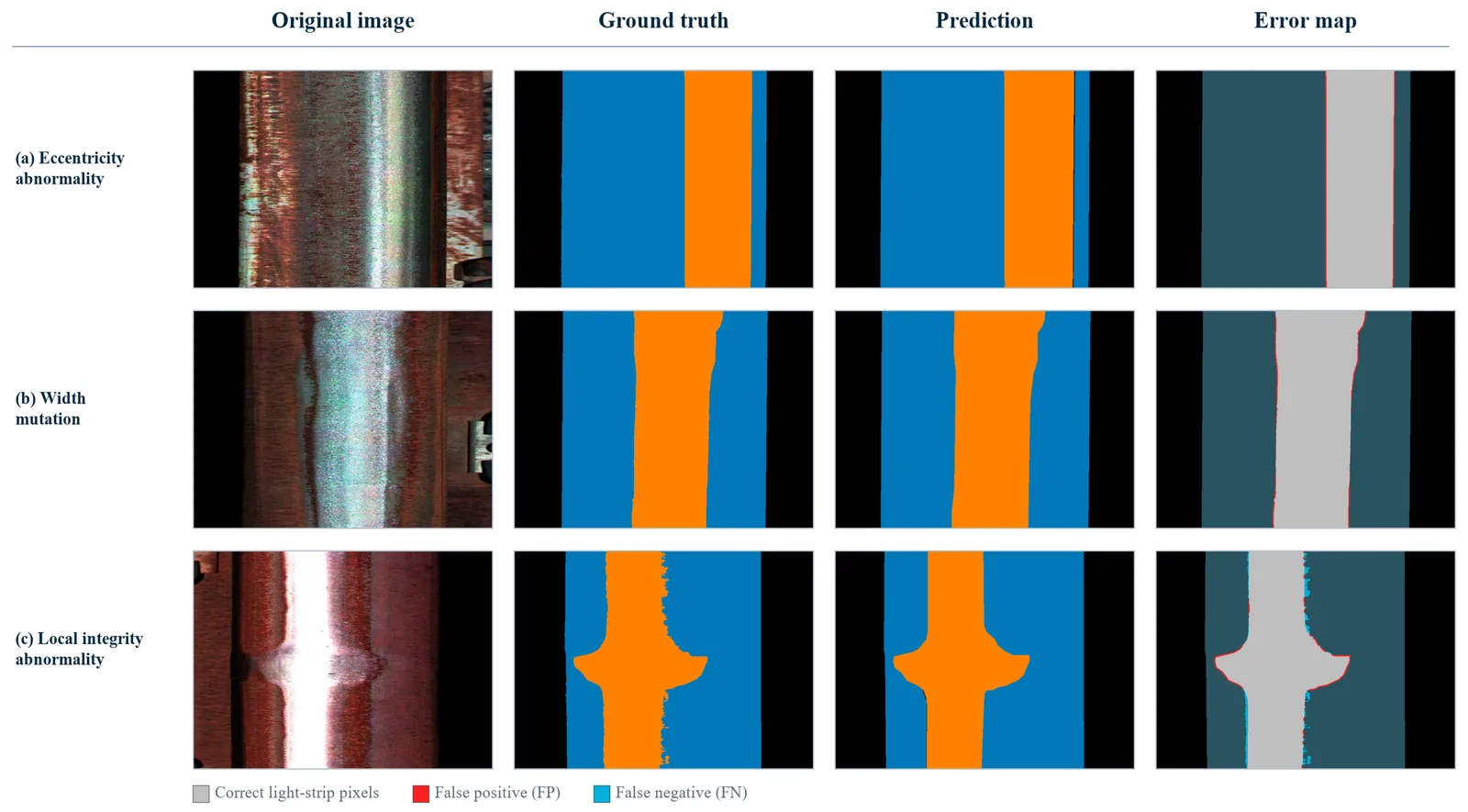
Typical examples of the three-rail light-strip abnormality categories. Rows (**a**–**c**) show eccentricity abnormality, width mutation and local integrity abnormality, respectively. The columns show the original image, Ground truth, improved SegFormer prediction and light-strip error map. Red and cyan denote false-positive and false-negative light-strip pixels, respectively.

**Figure 9:**
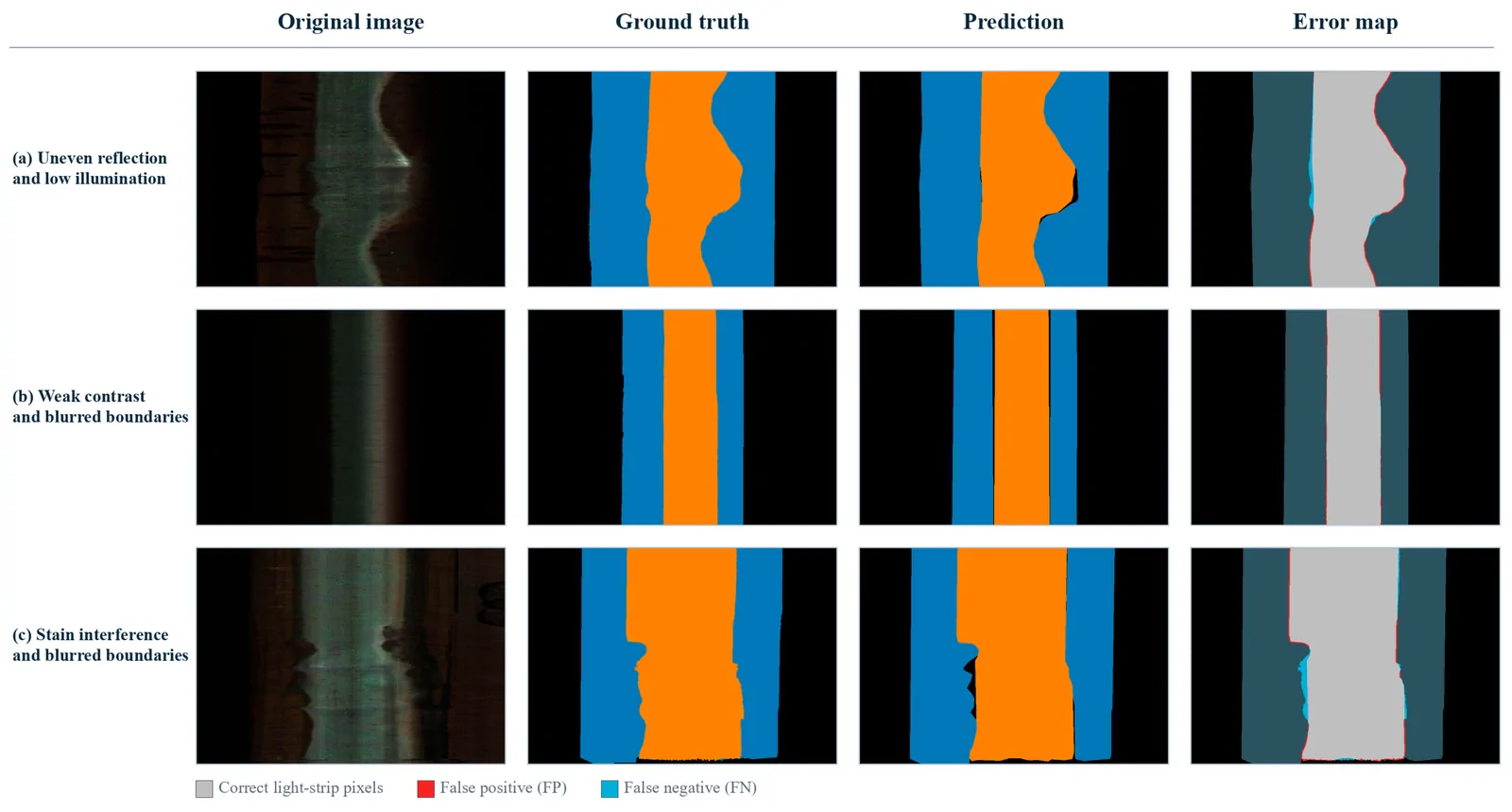
Challenging rail light-strip samples and residual segmentation errors. Rows (**a**–**c**) show uneven reflection with low illumination, weak contrast with blurred boundaries, and stain interference with blurred boundaries, respectively. The columns show the original image, ground truth, improved SegFormer prediction and light-strip error map. Red and cyan denote false-positive and false-negative light-strip pixels, respectively.

Figure 8 and Figure 9 examine representative abnormal morphologies and adverse imaging conditions. Each row compares the original image, ground truth, improved SegFormer prediction and light-strip error map. Red pixels are false positives, cyan pixels are false negatives, and gray pixels are correctly segmented light-strip regions.

**Figure 10:**
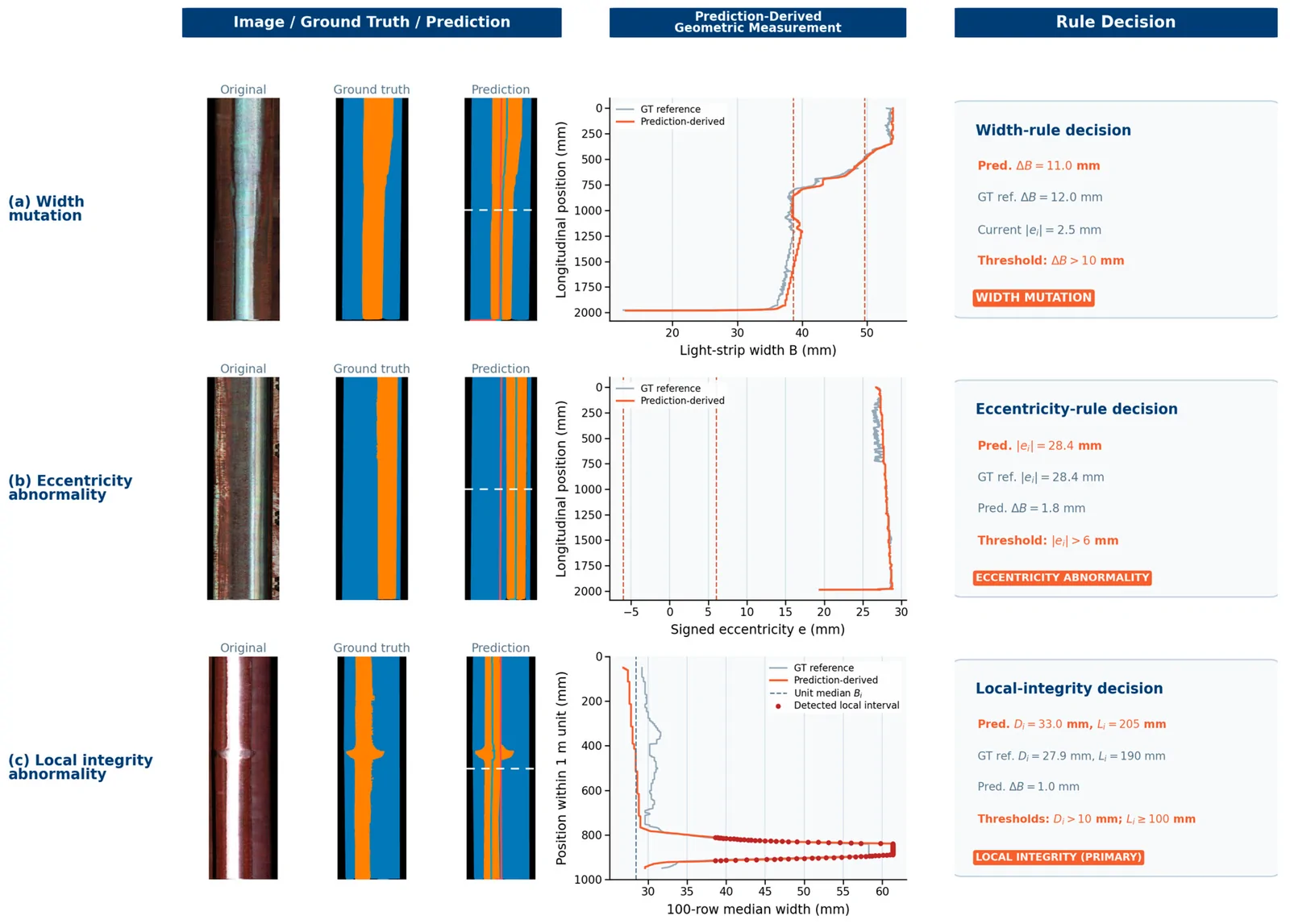
Representative geometric-rule abnormality decisions. Rows (**a**–**c**) show width mutation, eccentricity abnormality and local integrity abnormality, respectively. The columns compare the original image, Ground truth, prediction with rail-head and light-strip centerlines, prediction-derived geometric measurements and the triggered rule. Ground truth is shown only as an offline validation reference and is not involved in rule inference. The white dashed line marks a boundary between adjacent 1 m detection units.

Figure 10 traces the decision process for three representative validation cases. For L215, the prediction-derived width change is 11.0 mm and exceeds the 10 mm threshold, while the current-unit absolute eccentricity is 2.5 mm. For L732, the prediction-derived absolute eccentricity is 28.4 mm and exceeds the 6 mm limit, whereas the adjacent-unit width change is only 1.8 mm. For R1517, the maximum local width deviation is 33.0 mm, and its merged longitudinal extent is 205 mm, satisfying the local-integrity rule. Eccentricity abnormality describes the light-strip position relative to the rail-head centerline. It may coexist with a local contour abnormality, while the manually audited primary label is used for per-type statistics. All geometric measurements and rule decisions shown in the figure are calculated from the predicted masks. Ground-truth measurements are presented only as offline validation references. They do not participate in rule inference.

### 4.4. Computational Complexity and Deployment Efficiency

Deployment efficiency was evaluated at 512 × 994 pixels on the same RTX 3090 platform. With batch size 1, the MiT-B2 baseline has 27.4 M parameters. It requires 121.1 giga floating-point operations (GFLOPs). Its floating-point 32-bit (FP32) model is 109.6 MB, and its throughput is 38.7 frames/s. Peak GPU memory is 7.11 GB. The improved model has 27.9 M parameters, 122.5 GFLOPs, a 111.6 MB model, 38.3 frames/s throughput and 7.17 GB peak memory.

Geometric post-processing takes 2.58 ms per image. The complete improved pipeline therefore requires approximately 28.69 ms per image, corresponding to 34.9 frames/s. The boundary branch increases parameter count by 1.8%, computation by 1.2% and peak memory by 0.8%, while reducing throughput by about 1.0%. Focal Loss is used only during training and has no inference cost. The complete complexity results are summarized in Table 4.

## 5. Discussion

The method follows a perception-to-measurement process. SegFormer produces rail-head and light-strip masks, after which the rule module returns mileage, eccentricity, width change and local-integrity result. These outputs answer whether an abnormality is present, where it occurs and which geometric condition is triggered. This differs from a generic defect detector that returns only a class or bounding box.

The experiments also show why segmentation quality alone is insufficient. Higher mIoU does not automatically produce the highest precision after rule analysis. Boundary and centerline errors have a direct effect on geometric measurements. The BF1, CL-MAE and W-MAE results therefore complement mIoU and explain the improvement in abnormality recall and F1-score.

The rail-head mask is used as the geometric reference rather than treating the light strip as an isolated bright object. This reduces dependence on local intensity and makes each output traceable to a measured center shift, width change or local contour deformation. The indicators support targeted review but do not replace maintenance assessment.

Several limitations remain. The 6 mm absolute eccentricity and 10 mm width-mutation thresholds are engineering mappings whose transferability to different lines, speeds, illumination and camera installations requires external validation. The proprietary dataset limits independent reproduction. Reflection, stains and blurred boundaries can still cause localized false-positive and false-negative pixels, as shown in Figure 9. Table 3 shows that local integrity abnormality remains the most error-prone category. Reflection, stains and weak boundaries can disturb its local area and contour measurements.

Future work will evaluate the method on independent railway sections and the target onboard platform. It will also investigate normalized or adaptive thresholds, temporal consistency across consecutive images and the association between geometric indicators and verified maintenance records.

## 6. Conclusions

This study developed a rail light-strip abnormality analysis method that combines boundary-enhanced SegFormer segmentation with rail-head-constrained geometric rules. The method jointly extracts the rail head and light strip. Consecutive masks are ordered by corrected mileage and divided into 1 m units. For each triggered unit, the output record reports the start-end mileage interval, absolute eccentricity, width change and local-integrity result. This provides an explicit abnormality decision and identifies the geometric condition that triggered it.

The improved model achieves 95.67% mIoU, 65.75% BF1, 1.11 px CL-MAE and 4.50 px W-MAE. Among 876 ground-truth-positive 1 m units, 845 are correctly detected. Abnormality recall, precision and F1-score are 96.46%, 89.23% and 92.70%, respectively. Post-processing takes 2.58 ms per image, and the end-to-end throughput is 34.9 frames/s. These results support the use of the generated records for targeted manual review.

The reported interval is intended to guide targeted manual review rather than serve as an automatic maintenance grade. The longitudinal sampling and engineering rules used in this study define the localization resolution as 1 m. The available tests are limited to the inspected lines, acquisition configuration and RTX 3090 platform. Future work will evaluate independent railway sections and the target onboard system. It will also examine adaptive thresholds, temporal consistency across consecutive images and associations with verified maintenance records.

## Figures and Tables

**Table 1:** Ablation study with class-wise segmentation, boundary-quality and geometric-measurement metrics. BG: background; RH: rail head; LS: light strip; BF1: boundary F1 score computed with a 3-pixel tolerance; CL-MAE: centerline mean absolute error; W-MAE: width mean absolute error. Lower CL-MAE and W-MAE values indicate better performance.

Variant	BG IoU	RH IoU	LS IoU	mIoU	BF1	CL-MAE	W-MAE
SegFormer	95.00%	89.70%	93.88%	92.86%	64.72%	1.80 px	5.61 px
+Boundary enhancement	96.10%	91.60%	94.12%	93.94%	65.43%	1.15 px	4.63 px
+Focal Loss	96.35%	91.25%	94.76%	94.12%	64.96%	1.38 px	5.27 px
+Boundary enhancement + Focal Loss	97.18%	93.52%	96.31%	95.67%	65.75%	1.11 px	4.50 px

**Table 2:** Abnormality analysis performance based on different segmentation models and the same geometric rules.

Model	mIoU	Recall	Precision	F1-Score
DeepLab v3+	90.52%	87.67%	85.91%	86.78%
Segmenter	91.31%	91.10%	85.81%	88.38%
OCRNet	91.67%	93.15%	88.31%	90.67%
BiSeNetV2	92.12%	93.61%	90.11%	91.83%
SegFormer	92.86%	94.98%	88.51%	91.63%
Improved SegFormer	95.67%	96.46%	89.23%	92.70%

**Table 3:** Per-type abnormality-analysis results on the validation set. Ground-truth-positive units are mutually exclusive primary abnormality labels. Bold values indicate the overall results.

Abnormality Type	Ground-Truth Positive Units	TP	FP	FN	Recall	Precision	F1-Score
Width mutation	438	430	42	8	98.17%	91.10%	94.51%
Eccentricity abnormality	292	279	38	13	95.55%	88.01%	91.63%
Local integrity abnormality	146	136	22	10	93.15%	86.08%	89.47%
**Overall**	**876**	**845**	**102**	**31**	**96.46%**	**89.23%**	**92.70%**

**Table 4:** Model complexity and deployment efficiency at 512 × 994 pixels. Frames per second (FPS) reports model inference throughput with batch size 1; post-processing time is measured separately.

Model	Params	FLOPs	Model Size	FPS	Peak Memory	Post-Process
SegFormer-B2	27.4 M	121.1 G	109.6 MB	38.7	7.11 GB	2.58 ms
Improved SegFormer	27.9 M	122.5 G	111.6 MB	38.3	7.17 GB	2.58 ms

## Data Availability

The data presented in this study are available from the corresponding author upon reasonable request and subject to authorization and approval for access to proprietary railway inspection data.

## References

[1] Du Y., Zhang X., Gao Y., Nan Z. (2024). RSDNet: A New Multiscale Rail Surface Defect Detection Model. Sensors.

[2] Faghih-Roohi S., Hajizadeh S., Nunez A., Babuska R., De Schutter B. (2016). Deep Convolutional Neural Networks for Detection of Rail Surface Defects. Proceedings of the International Joint Conference on Neural Networks.

[3] Gan J., Li Q., Wang J., Yu H. (2017). A Hierarchical Extractor-Based Visual Rail Surface Inspection System. IEEE Sens. J..

[4] Gibert X., Patel V.M., Chellappa R. (2015). Robust Fastener Detection for Autonomous Visual Railway Track Inspection. Proceedings of the IEEE Winter Conference on Applications of Computer Vision.

[5] Mauz F., Wigger R., Gota A.-E., Kuffa M. (2024). Automatic Detection of the Running Surface of Railway Tracks Based on Laser Profilometer Data and Supervised Machine Learning. Sensors.

[6] Xia Y., Han S.W., Kwon H.J. (2023). Image Generation and Recognition for Railway Surface Defect Detection. Sensors.

[7] Ming G., Zhou B., Luo X., Ling R., Zhou M. (2023). Rail Surface Defect Detection Method Based on Deep Learning Method with 3D Range Image. Advances in Frontier Research on Engineering Structures.

[8] Wang X., Xu X., Mei X., Guo X. (2025). Localization and Pixel-Confidence Network for Surface Defect Segmentation. Sensors.

[9] Leiñena A., Saiz F., Barandiaran I. (2024). Latent Diffusion Models to Enhance the Performance of Visual Defect Segmentation Networks in Steel Surface Inspection. Sensors.

[10] Zhong Y., Chen G. (2024). Rail Surface Defect Detection Based on Dual-Path Feature Fusion. Electronics.

[11] Gosiewska A., Baran Z., Baran M., Rutkowski T. (2023). Seeking a Sufficient Data Volume for Railway Infrastructure Component Detection with Computer Vision Models. Sensors.

[12] Long J., Shelhamer E., Darrell T. (2015). Fully Convolutional Networks for Semantic Segmentation. Proceedings of the IEEE Conference on Computer Vision and Pattern Recognition.

[13] Ronneberger O., Fischer P., Brox T. (2015). U-Net: Convolutional Networks for Biomedical Image Segmentation. Proceedings of the Medical Image Computing and Computer-Assisted Intervention.

[14] Zhao H., Shi J., Qi X., Wang X., Jia J. (2017). Pyramid Scene Parsing Network. Proceedings of the IEEE Conference on Computer Vision and Pattern Recognition.

[15] Chen L.C., Zhu Y., Papandreou G., Schroff F., Adam H. (2018). Encoder-Decoder with Atrous Separable Convolution for Semantic Image Segmentation. Proceedings of the European Conference on Computer Vision.

[16] Zheng S., Lu J., Zhao H., Zhu X., Luo Z., Wang Y., Fu Y., Feng J., Xiang T., Torr P.H.S. (2021). Rethinking Semantic Segmentation from a Sequence-to-Sequence Perspective with Transformers. Proceedings of the IEEE/CVF Conference on Computer Vision and Pattern Recognition.

[17] Strudel R., Garcia R., Laptev I., Schmid C. (2021). Segmenter: Transformer for Semantic Segmentation. Proceedings of the IEEE/CVF International Conference on Computer Vision.

[18] Liu Z., Lin Y., Cao Y., Hu H., Wei Y., Zhang Z., Lin S., Guo B. (2021). Swin Transformer: Hierarchical Vision Transformer Using Shifted Windows. Proceedings of the IEEE/CVF International Conference on Computer Vision.

[19] Xie E., Wang W., Yu Z., Anandkumar A., Alvarez J.M., Luo P. (2021). SegFormer: Simple and Efficient Design for Semantic Segmentation with Transformers. Advances in Neural Information Processing Systems.

[20] Takikawa T., Acuna D., Jampani V., Fidler S. Gated-SCNN: Gated Shape CNNs for Semantic Segmentation. Proceedings of the IEEE/CVF International Conference on Computer Vision.

[21] Yuan Y., Xie J., Chen X., Wang J. (2020). SegFix: Model-Agnostic Boundary Refinement for Segmentation. Computer Vision-ECCV 2020.

[22] Cheng B., Girshick R., Dollar P., Berg A.C., Kirillov A. Boundary IoU: Improving Object-Centric Image Segmentation Evaluation. Proceedings of the IEEE/CVF Conference on Computer Vision and Pattern Recognition.

[23] Cheng B., Misra I., Schwing A.G., Kirillov A., Girdhar R. (2022). Masked-Attention Mask Transformer for Universal Image Segmentation. Proceedings of the IEEE/CVF Conference on Computer Vision and Pattern Recognition.

[24] Yuan Y., Chen X., Wang J. (2020). Object-Contextual Representations for Semantic Segmentation. Proceedings of the European Conference on Computer Vision.

[25] Yu C., Gao C., Wang J., Yu G., Shen C., Sang N. (2021). BiSeNet V2: Bilateral Network with Guided Aggregation for Real-Time Semantic Segmentation. Int. J. Comput. Vis..

[26] Zhu Y., Xiao N. (2024). Simple Scalable Multimodal Semantic Segmentation Model. Sensors.

[27] Shu X., Zhao X. (2024). Multi-Resolution Learning and Semantic Edge Enhancement for Super-Resolution Semantic Segmentation of Urban Scene Images. Sensors.

[28] Zhang X., Chen X., Gao X. (2024). Semantic Guidance Fusion Network for Cross-Modal Semantic Segmentation. Sensors.

[29] Kirillov A., Mintun E., Ravi N., Mao H., Rolland C., Gustafson L., Xiao T., Whitehead S., Berg A.C., Lo W.Y. (2023). Segment Anything. Proceedings of the IEEE/CVF International Conference on Computer Vision.

[30] Lin T.Y., Goyal P., Girshick R., He K., Dollár P. (2017). Focal Loss for Dense Object Detection. Proceedings of the IEEE International Conference on Computer Vision.

[31] (2021). Technical Specification for Rail Grinding and Maintenance of Urban Rail Transit.

[32] National Railway Administration (2023). Rules for Maintenance of High-Speed Railway Lines, Guo Tie She Bei Jian Gui [2023] No. 15.

[33] China State Railway Group Co., Ltd., Engineering and Electrical Department (2021). Railway Line Maintenance and Repair.

[34] Russell B.C., Torralba A., Murphy K.P., Freeman W.T. (2008). LabelMe: A Database and Web-Based Tool for Image Annotation. Int. J. Comput. Vis..

[35] Everingham M., Van Gool L., Williams C.K.I., Winn J., Zisserman A. (2010). The Pascal Visual Object Classes Challenge. Int. J. Comput. Vis..

[36] Deng J., Dong W., Socher R., Li L.J., Li K., Fei-Fei L. (2009). ImageNet: A Large-Scale Hierarchical Image Database. Proceedings of the IEEE Conference on Computer Vision and Pattern Recognition.

[37] Sutskever I., Martens J., Dahl G., Hinton G. (2013). On the Importance of Initialization and Momentum in Deep Learning. Proceedings of the International Conference on Machine Learning.

